# Characterization of the *Candida glabrata* Transcription Factor CgMar1: Role in Azole Susceptibility

**DOI:** 10.3390/jof8010061

**Published:** 2022-01-07

**Authors:** Pedro Pais, Mónica Galocha, Raquel Califórnia, Romeu Viana, Mihaela Ola, Michiyo Okamoto, Hiroji Chibana, Geraldine Butler, Miguel C. Teixeira

**Affiliations:** 1 Department of Bioengineering, Instituto Superior Técnico, Universidade de Lisboa, 1049-001 Lisboa, Portugal; pedrohpais@tecnico.ulisboa.pt (P.P.); monicagalocha@tecnico.ulisboa.pt (M.G.); raquelsc1995@hotmail.com (R.C.); romeuviana@tecnico.ulisboa.pt (R.V.); 2 iBB—Institute of Bioengineering and Biosciences, Biological Sciences Research Group, Instituto Superior Técnico, 1049-001 Lisboa, Portugal; 3 Associate Laboratory i4HB—Institute for Health and Bioeconomy at Instituto Superior Técnico, Universidade de Lisboa, 1049-001 Lisboa, Portugal; 4 School of Biomedical and Biomolecular Sciences, Conway Institute, University College Dublin, Dublin 4, Ireland; mihaela.ola@ucdconnect.ie (M.O.); gbutler@ucd.ie (G.B.); 5 Medical Mycology Research Center (MMRC), Chiba University, Chiba 263-8522, Japan; m-sato_okamoto@chiba-u.jp (M.O.); chibana@faculty.chiba-u.jp (H.C.)

**Keywords:** *Candida glabrata*, azole resistance, transcription regulatory networks, CgMar1, transcriptomics, CgRsb1

## Abstract

The prevalence of antifungal resistance in *Candida glabrata*, especially against azole drugs, results in difficult-to-treat and potentially life-threatening infections. Understanding the molecular basis of azole resistance in *C*. *glabrata* is crucial to designing more suitable therapeutic strategies. In this study, the role of the transcription factor encoded by ORF *CAGL0B03421g*, here denominated as CgMar1 (Multiple Azole Resistance 1), in azole susceptibility was explored. Using RNA-sequencing, CgMar1 was found to regulate 337 genes under fluconazole stress, including several related to lipid biosynthesis pathways. In this context, CgMar1 and its target *CgRSB1*, encoding a predicted sphingoid long-chain base efflux transporter, were found to contribute to plasma membrane sphingolipid incorporation and membrane permeability, decreasing fluconazole accumulation. CgMar1 was found to associate with the promoter of *CgRSB1*, which contains two instances of the CCCCTCC consensus, found to be required for *CgRSB1* activation during fluconazole stress. Altogether, a regulatory pathway modulating azole susceptibility in *C*. *glabrata* is proposed, resulting from what appears to be a neofunctionalization of a Hap1-like transcription factor.

## 1. Introduction

*Candida* species constitute the most common cause of fungal infections, representing the fourth leading cause of nosocomial bloodstream infections in the USA [1,2,3]. *C*. *albicans* is the most isolated species, but the prevalence of non-*albicans* infections has been steadily increasing. *Candida glabrata* ranks as the second or third most commonly isolated pathogenic *Candida* spp. depending on the geographical region [4,5,6], at least partially due to its ability to rapidly acquire azole resistance. One reason for the emergence of resistance in *C*. *glabrata* is related to its haploid nature, which facilitates acquisition of mutations in resistance genes.

Azole antifungals are widely used in clinical practice as both treatment and prophylaxis of fungal infections [7]. Azoles inhibit ergosterol biosynthesis by targeting the Erg11 enzyme, leading to loss of plasma membrane properties (e.g., fluidity, stability, structure, asymmetry, and function) by ergosterol depletion and incorporation of the toxic sterol dimethylcholesta-8,24(28)-dien-3β,6α-diol (DMCDD) in the membrane [8,9]. In *C*. *glabrata*, the transcription factors (TFs) CgUpc2a and CgUpc2b are major regulators of ergosterol biosynthesis, while in *C*. *albicans*, this pathway is controlled by a single TF (CaUpc2) [10,11,12]. Azole resistance in *Candida* spp. is typically mediated by TFs commonly referred to as azole stress response regulators. This is the case of CgPdr1 in *C*. *glabrata* and CaTac1 in *C*. *albicans*, activators of multidrug resistance transporters (MDR) from the ATP-Binding Cassette (ABC), such as *CDR1* and *CDR2* [13,14,15,16,17,18,19,20]. Additionally, *C*. *albicans* also carries the TF CaMrr1, which activates the *MDR1* transporter from the Major Facilitator Superfamily (MFS) [21,22].

Here, the role of the *C*. *glabrata* TF encoded by the *CAGL0B03421g* ORF, here designated as CgMar1 (Multiple Azole Resistance 1), in azole susceptibility is analyzed. Its role in the transcriptome-wide response to fluconazole is assessed, leading to the elucidation of its role in modulating azole susceptibility, dependent on membrane sphingolipid incorporation, membrane permeability, and intracellular drug accumulation.

## 2. Materials and Methods

### 2.1. Plasmids, Strains, and Growth Media

The plasmid pGREG576 was obtained from the Drag&Drop collection [23]. The plasmid pV1382 [24] was obtained from Addgene (Addgene plasmid# 111436).

All *C*. *glabrata* strains used in this study are presented in Table 1. The *C*. *glabrata* parental strain KUE100 [25] and derived single deletion mutants were batch cultured at 30 °C, with orbital agitation (250 rpm) in basal medium (BM), with the following composition (per liter): 1.7 g of yeast nitrogen base without amino acids or NH4+ (Difco, England, UK), 20 g of glucose (Merck, Darmstadt, Germany), and 2.65 g of (NH4)2SO4 (Merck). KUE100::URA- and the derived KUE100_*Δcgmar1*::URA- strains, harboring pGREG576-derived plasmids, were grown in BM. The *S*. *cerevisiae* strain BY4741 (MATa, ura3Δ0, leu2Δ0, his3Δ1, and met15Δ0) was obtained from Euroscarf and grown in Yeast extract-Peptone-Dextrose (YPD) medium, with the following composition (per liter): 20 g of glucose (Merck), 20 g of Peptone (Merck), and 10 g of Yeast extract (Merck). Besides the above-indicated ingredients, the solid media contained 20 g/L of agar (Iberagar, Barreiro, Portugal).

### 2.2. Disruption of the C. glabrata CgHAP1, CgMAR1, and CgRSB1 Genes (ORFs CAGL0K05841g, CAGL0B03421g, and CAGL0L10142g, Respectively)

The deletion of the *C*. *glabrata* genes addressed in this study was carried out in the parental strain KUE100 using the method described by Ueno et al. [28]. Genes of interest were replaced by a DNA cassette including the *CgHIS3* gene, through homologous recombination. The PCR primers used to generate the replacement cassette for each gene and the primers used for PCR confirmation of gene deletion are present in Table S1. The pHIS906 plasmid including *CgHIS3* was used as a template, and transformation was performed as described previously [25].

### 2.3. Cloning of the C. glabrata CgMAR1 Gene (ORF CAGL0B03421g)

The pGREG576 plasmid from the Drag&Drop collection was used as described before to clone and express the *C*. *glabrata* ORF *CAGL0B03421g* [26,29,30,31]. pGREG576 was acquired from Euroscarf and contains a galactose inducible promoter (*GAL1*); the yeast selectable marker *URA3*; and the *GFP* gene, encoding a Green Fluorescent Protein (GFPS65T), which allows for monitoring of the expression and subcellular localization of the cloned fusion protein. *CgMAR1* DNA was generated by PCR, using genomic DNA extracted from the sequenced CBS138 *C*. *glabrata* strain. Besides a region with homology to the first 20 and last 22 nucleotides of the *CgMAR1* coding region (italic), the designed primers contain nucleotide sequences with homology to the cloning site flanking regions of the pGREG576 vector (underlined). The amplified fragments were co-transformed into the parental *S*. *cerevisiae* strain BY4741 with the pGREG576 vector, previously cut with the restriction enzyme SalI to obtain the pGREG576_*CgMAR1* plasmid. Since the *GAL1* promoter only allows for a very low expression of downstream genes in *C*. *glabrata*, the *GAL1* promoter present in the pGREG576_*CgMAR1* plasmid was replaced by the constitutive *PDC1 C*. *glabrata* promoter [32], giving rise to the pGREG576_PDC1_*CgMAR1* plasmid. The *PDC1* promoter DNA was generated by PCR, using genomic DNA extracted from the sequenced CBS138 *C*. *glabrata* strain. Besides a region with homology to 22 nucleotides in the beginning and the last 22 nucleotides of the 1000 bp upstream region of the *PDC1* coding sequence (italic), the designed primers contain nucleotide sequences with homology to the cloning site flanking regions of the pGREG576 vector (underlined). The amplified fragment was co-transformed into the parental strain BY4741 with the pGREG576_*CgMAR1* plasmid, previously cut with AscI and NotI restriction enzymes to remove the *GAL1* promoter, to generate the pGREG576_PDC1_*CgMAR1* plasmid. The recombinant plasmids pGREG576_*CgMAR1* and pGREG576_PDCI_*CgMAR1* were obtained through homologous recombination in *S*. *cerevisiae* and verified by DNA sequencing. All primers used are presented in Table S1.

### 2.4. Disruption of C. glabrata CgURA3 Gene (ORF CAGL0I03080g)

The deletion of the *C*. *glabrata URA3* gene encoded by ORF *CAGL0I03080g* was carried out in the *Δcgmar1* mutant as described before [26] using the CRISPR-Cas9 system from Vyas et al. [24]. Briefly, a *CgURA3* gRNA sequence selected from the resources made available by Vyas et al. [24] was cloned in the pV1382 plasmid, previously linearized with the restriction enzyme BsmBI (New England Biolabs, Ipswich, MA, USA). The *CgURA3* gRNA was obtained by oligonucleotide annealing and the product ligated into the previously linearized pV1382 plasmid to obtain the pV1382_*CgURA3* vector. The construct was verified by DNA sequencing. The plasmid was transformed into the *Δcgmar1* mutant and cells were directly plated on 5-Fluoroorotic acid (5-FOA) to select for URA- cells. Sequential passages in non-selective medium (YPD) were performed to avoid detrimental effects of further Cas9 expression, and *CgURA3* loss of function was further confirmed by the inability to grow in medium without uracil. The introduction of pGREG576-derived plasmids (containing a *URA3* selection marker) in the edited strains was able to rescue the growth impairment in the absence of uracil. All primers used are presented in Table S1.

### 2.5. Antifungal Susceptibility Assays

KUE100 *C*. *glabrata* and the derived deletion mutant cell suspensions used to inoculate agar plates were mid-exponential cells grown in BM, as were the KUE100::URA- and the derived deletion mutant KUE100_*Δcgmar1*::URA-. Cells were grown until a culture OD600nm of 0.5 ± 0.05 was reached and then diluted in sterile water to obtain suspensions with OD600nm = 0.05 ± 0.005. These cell suspensions and subsequent dilutions (1:5; 1:25) were applied as 4 µL spots onto the surface of solid BM plates supplemented with distinct chemical stress concentrations. The tested drugs included the following compounds, used in the following specified concentration ranges: the azole antifungal drugs ketoconazole (10 to 60 mg/L), fluconazole (100 to 250 mg/L), miconazole (0.10 to 0.50 mg/L), itraconazole (15 to 30 mg/L), and clotrimazole (2.5 to 15 mg/L).

MIC assays were performed in 96-well plates containing RPMI-1640 2% glucose medium with the appropriate drug concentrations as described previously and according to EUCAST guidelines [29].

### 2.6. Total RNA Extraction

*C*. *glabrata* strains KUE100 and KUE100_*∆cgmar1* were grown in BM until mid-exponential phase. Subsequently, the cells were transferred to fresh medium (control), or fresh medium containing 150 mg/L of fluconazole and harvested after 1h of incubation. Total RNA was isolated using an Ambion Ribopure-Yeast RNA kit according to manufacturer’s instructions. The chosen concentration of fluconazole is higher than the MIC attained by microdilution testing. Resistance measurements can vary according to distinct experimental conditions, such as cell density/inoculum size, which has been demonstrated to impact inhibitory concentrations of antifungal drugs (i.e., higher cell densities result in lower drug susceptibility) [33]. The selected concentration was found to be inhibitory under the conditions used for cell growth in this study.

### 2.7. Library Preparation and Gene Expression Analysis

Strand-specific RNA-seq library preparation and sequencing was carried out as a paid service by the NGS core from Oklahoma Medical Research Foundation, Oklahoma City, Oklahoma. Libraries were generated using the Illumina Truseq Stranded Total RNA library prep kit with ribosomal depletion via RiboZero Gold according to the manufacturer’s protocol. Raw data are available at GEO under accession number: GSE163158. Raw data for the wild type were obtained concurrently and has been previously submitted [29]. Bioinformatics analysis was performed as described previously [26,29]. Briefly, sample reads were trimmed using Skewer (v0.2.2) [34] and aligned to the *C*. *glabrata* CBS138 reference genome, obtained from the *Candida* Genome Database (CGD), using TopHat (v2.1.1) [35] with the parameters −p 12 (number of threads), −g 1 (maximum amount of times that a read can be mapped to the genome), −b2-very-sensitive (preset option), and −library-type fr-firststrand (to account for strand specificity). HTSeq (v0.7.1) [36] was used to count the mapped reads per gene. Differentially expressed genes were identified using DESeq2 [37] with an adjusted *p*-value threshold of 0.05 and a log2 fold change greater than 0.5 or less than −0.5. Default parameters in DESeq2 were used.

### 2.8. NBD-DHS Subcellular Localization Assessment

*C*. *glabrata* cell suspensions from strains KUE100, KUE100_*∆cgmar1*, and KUE100_*∆cgrsb1* were prepared in BM until a standard culture OD600nm of 0.5 ± 0.05 was reached. C6 NBD-dihydrosphingosine (NBD-DHS; 1 mg/mL in methanol; Santa Cruz Biotechnology, Santa Cruz, CA, USA) was added to 1 mL of 4 × 10^7^ cells/mL to a final concentration of 5 µM, and cell suspensions were incubated in the dark with orbital agitation (30 min, 250 rpm). Cells exposed to C6 NBD-DHS were centrifuged (17,500× *g* for 5 min), washed twice, and resuspended in PBS buffer to final 10^7^ cells/mL aliquots. NBD fluorescence was detected by fluorescence microscopy in a Zeiss Axioplan microscope (Carl Zeiss MicroImaging, Oberkochen, Germany), using excitation and emission wavelengths of 395 and 509 nm, respectively. Fluorescence images were captured using a cooled Zeiss Axiocam 503 color (Carl Zeiss Microscopy, Oberkochen, Germany). Quantification of NBD-DHS signal localization to cell membranes was performed by accessing a minimum of 100 cells per strain.

### 2.9. Plasma Membrane Permeability

Plasma membrane permeability was assessed by the passive uptake of propidium iodide (PI; 20 mM in DMSO, Invitrogen, Waltham, MA, USA) as described previously [26,29]. *C*. *glabrata* cell suspensions from strains KUE100, KUE100_*∆cgmar1*, and KUE100_*∆cgrsb1* were prepared in BM until a standard culture OD600nm of 0.5 ± 0.05 was reached and transferred to the same medium with or without 150 mg/L of fluconazole. After 1 h of incubation, PI was added to 1 mL of 4 × 10^7^ cells/mL to a final concentration of 20 µM, and cell suspensions were incubated in the dark with orbital agitation (15 min, 250 rpm). Cells exposed to PI were centrifuged (17,500× *g* for 5 min), washed twice, resuspended in PBS buffer, and pooled to final 10^7^ cells/mL aliquots. PI fluorescence was detected by fluorescence microscopy in a Zeiss Axioplan microscope (Carl Zeiss MicroImaging) using excitation and emission wavelengths of 536 and 595 nm, respectively. Fluorescence images were captured using a cooled Zeiss Axiocam 503 color (Carl Zeiss Microscopy), and the images were analyzed with the ZEN lite software from ZEISS microscopy. Cell-to-cell fluorescence intensity was defined as the average of pixel-by-pixel intensity in the selected region of interest, and a minimum of 70 cells per strain were assessed. The fluorescence images were background corrected using dark-current images.

### 2.10. ^3^H-Fluconazole Accumulation Assays

^3^H-fluconazole transport assays were carried out as described before for other radiolabeled compounds [29,30,31,38]. The internal accumulation of fluconazole was determined by calculating the ratio between the radiolabeled fluconazole measured within the yeast cells and in the external medium (intracellular/extracellular). The parental strain KUE100 and the mutant strains KUE100_*Δcgmar1* and KUE100_*Δcgrsb1* were grown in BM until the mid-exponential phase and harvested by filtration. The cells were washed and resuspended in fresh BM to obtain dense cell suspensions (OD600nm = 0.5 ± 0.1, equivalent to approximately 1.57 mg (dry weight) mL^−1^). Readily, 0.1 µM of ^3^H-fluconazole (Moravek Inc., Brea, CA, USA; 1 mCi/mL) and 150 mg/L of unlabeled fluconazole were added to the cell suspensions. Incubation proceeded for an additional period of 30 min. The intracellular accumulation of labeled fluconazole was followed by filtering 200 µL of cell suspension at adequate time intervals through pre-wetted glass microfiber filters (Whatman GF/C, Merck, Darmstadt, Germany). The filters were washed with ice-cold TM buffer, and the radioactivity was measured in a Beckman LS 5000TD scintillation counter. Extracellular ^3^H-fluconazole was estimated by radioactivity assessment of 50 µL of the supernatant. Non-specific ^3^H-fluconazole adsorption to the filters and to the cells (less than 5% of the total radioactivity) was assessed and taken into consideration. To calculate the intracellular concentration of labeled fluconazole, the internal cell volume (Vi) of the exponential cells, grown in the absence of drug and used for accumulation assays, was considered constant and equal to 2.5 µL (mg dry weight)^−1^ [39].

### 2.11. In Silico Prediction of Overrepresented Sequences in CgMar1-Activated Promoters

The promoters (−1000 to −1 bp) upstream of the coding regions of genes for which their expressions were found to be activated by CgMar1 were retrieved using the “Retrieve Upstream Sequence” tool from PathoYeastract [40]. The obtained sequences were submitted to DREME (MEME suite) [41] for discovery of enriched sequences, using default parameters.

### 2.12. Cloning of the CgRSB1 Promoter and Site-Directed Mutagenesis

The pYPE354 plasmid was used as described before to clone and express the lacZ reporter gene [29]. pYEP354 contains the yeast selectable marker *URA3* and the bacterial selectable marker AmpR genes. *CgRSB1* promoter DNA was generated by PCR, using genomic DNA extracted from the sequenced CBS138 *C*. *glabrata* strain. The first primer contains a region with homology within the beginning of the *CgRSB1* promoter and a recognition site for the EcoRI restriction enzyme, flanked by additional bases. The second primer contains a region with homology within the end of the *CgRSB1* promoter and the beginning of the *CgRSB1* coding sequence and a recognition site for the PstI restriction enzyme, flanked by additional bases. The amplified fragment was ligated into the pYEP354 vector (T4 Ligase, New England Biolabs), previously cut with the same restriction enzymes, to obtain the pYEP354_*CgRSB1*prom_lacZ plasmid. The putative CgMar1 consensus in the *CgRSB1* promoter were mutated by site-directed mutagenesis. The designed primers contain two mutations within each potential consensus, resulting in the production of each mutated consensus by PCR amplification to obtain the pYEP354_mut_*CgRSB1*prom_lacZ plasmids. The original template was then degraded by DpnI digestion. All primers used are presented in Table S1.

### 2.13. Gene Expression Measurement by RT-PCR

The transcript levels of the lacZ reporter gene were determined by quantitative real-time PCR (RT-PCR). KUE100::URA- cells harboring the pYEP354_*CgRSB1*prom_lacZ or the pYEP354_mut_*CgRSB1*prom_lacZ plasmids were prepared in BM until a standard culture OD600nm of 0.5 ± 0.05 was reached and transferred to the same medium with or without 150 mg/L of fluconazole. After 1 h of incubation, the cells were harvested and immediately frozen at −80 °C until RNA extraction. For total RNA extraction, the hot phenol method was applied [42]. RT-PCR was performed as described elsewhere [26,29,30,31]. The synthesis of cDNA for real-time RT-PCR experiments, from total RNA samples, was performed using the MultiscribeTM reverse transcriptase kit (Applied Biosystems, Thermo Fisher Scientific, Waltham, MA, USA) and the 7500 RT-PCR Thermal Cycler Block (Applied Biosystems), following the manufacturer’s instructions. The quantity of cDNA for the following reactions was kept around 10 ng. The subsequent RT-PCR step was carried out using SYBR^®^ Green (NZYTech, Lisbon, Portugal) reagents with default parameters established by the manufacturer and the primers in Table S1. The *CgRDN25* gene transcript levels were used as an internal reference.

### 2.14. CgMar1 Chromatin Immunoprecipitation Assays Followed by RT-qPCR

KUE100::URA- cells harboring the pGREG576_PDC1_*CgMAR1* plasmid were grown to the mid-exponential phase in BM until 100 OD units were reached and then transferred to a fresh medium (control) or a fresh medium containing 150 mg/L fluconazole. After 1 h of incubation, the cells were cross-linked with 1% formaldehyde (15 min), and cross-linking was quenched with 340 mM glycine (10 min). The cells were collected, washed twice with cold PBS (pH 7.4), and frozen immediately at −80 °C. The cell pellets were lysed with 0.5 mm zirconia beads (Invitrogen) in lysis buffer (50 mM HEPES (pH 7.5), 140 mM NaCl, 1 mM EDTA, 1% Triton X-100, 0.5% NP-40, and 0.5 mM dithiothreitol (DTT)) by vortexing for 20 s and incubation on ice for 2 min (4 times). Cell debris was pelleted by centrifugation, and the lysate was collected. The lysates were sonicated four times for 2 min followed by 2 min on ice using a Bioruptor water bath sonicator. Sonicated lysates were cleared by centrifugation, and 5% of the sample was collected for use as the input (sonicated chromatin, not immunoprecipitated). For immunoprecipitation (IP), chromatin was incubated overnight at 4 °C (with rotation) with protein G SureBeads (Bio-Rad, Hercules, CA, USA) coupled with IgG anti-GFP antibody (catalog number sc-9996; Santa Cruz Biotechnology Inc.). The beads were washed according to the manufacturer’s instructions, and the samples were eluted at 65 °C for 20 min in Tris-EDTA (TE) buffer with 0.5% SDS. Input and immunoprecipitated samples were incubated overnight at 65 °C for cross-linking reversal. DNA was purified with the NZYGelPure kit (NZYTech). Quantification of a specific *CgRSB1* promoter region was performed by RT-PCR using 5 μL of the DNA preparation from each reaction mixture. The primers used are listed in Table S1. The immunoprecipitated samples were processed together with the input samples, and the amplification product was normalized to the total template used. Based on methods reported previously [29,43,44], the ratio of the immunoprecipitated promoter to the input (IP/input) was calculated, and ratios in fluconazole samples were compared with the ratio in the control samples at the specific locus of the *CgRSB1* promoter.

### 2.15. Used Primers

All primers used are presented in Table S1.

### 2.16. Statistical Analysis

All results represent the average of at least three independent experiments. Statistical analyses were performed using ANOVA with Tukey’s correction (comparisons of more than two groups) or t-tests with Welch’s correction (comparisons of two groups). Statistical analysis of RNA-seq data was performed using a Benjamini–Hochberg procedure. Significance levels are indicated in the figure legends (*, *p* < 0.05; **, *p* < 0.01; ***, *p* < 0.001; and ****, *p* < 0.0001).

## 3. Results

### 3.1. CgMar1 Affects Azole Susceptibility in Candida glabrata

In *C*. *albicans*, the TFs CaTac1 and CaMrr1 are the main azole resistance regulators, while in *C*. *glabrata*, only CgPdr1 is recognized as taking this role. We searched for a possible CaMrr1 counterpart in *C*. *glabrata* by first assessing protein sequence similarity: the protein encoded by the uncharacterized *C*. *glabrata* ORF *CAGL0B03421g* shows the highest similarity hit for *C*. *albicans* Mrr1, although with limited identity (E-value = 10^−20^; 25.55% identity). Reciprocal blast attains CaMrr1 as the best hit homolog for the protein encoded by ORF *CAGL0B03421g* (E-value = 10^−18^; 25.55% identity). Interestingly, the ORF *CAGL0B03421g* has a paralog designated *CgHAP1* (ORF *CAGL0K05841g*) and the two proteins share 38.5% identity. Syntenic context [45,46] reveals that both *CAGL0B03421g* and *CAGL0K05841g* are paralogs of *S*. *cerevisiae HAP1*, resulting from genome duplication and subsequent loss of one in *S*. *cerevisiae*. Syntenic analysis also revealed the *HAP1* homolog in *C*. *albicans* to be TF *CaZCF20* (and not *CaMRR1*) despite reduced sequence similarity (Figure S1A). On the other hand, *C*. *glabrata CAGL0B03421g*/CgHap1 and *C*. *albicans* Mrr1 share some degree of sequence similarity on the DNA binding domain and a regulatory middle homology domain (Figure S1B).

**Figure 1:**
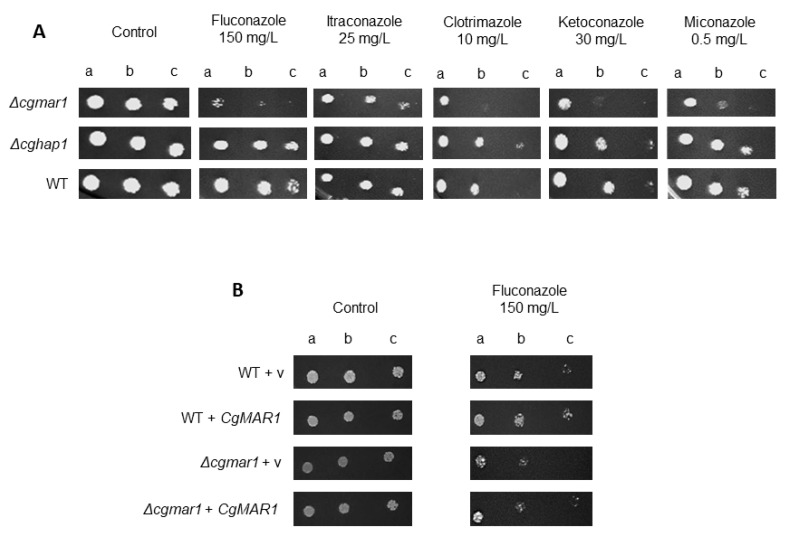
*CgMAR1* (ORF *CAGL0B03421g*) mediates tolerance to fluconazole and other azoles. (**A**) Comparison of spot growth assays of the KUE100 *C*. *glabrata* wild-type and derived KUE100_*Δcghap1* and KUE100_*Δcgmar1* deletion mutants in the presence of azole antifungals. (**B**) Comparison of spot growth assays of the KUE100::URA- *C*. *glabrata* wild-type and derived KUE100_*Δcgmar1*::URA- deletion mutant harboring the pGREG576 cloning vector or the pGREG576_PDC1_*CgMAR1* expression plasmid in the presence of fluconazole. The inocula were prepared as described in the Materials and Methods section. The cell suspensions used to prepare the spots were 1:5 (b) and 1:25 (c) dilutions of the cell suspensions used in (a). The images displayed are representative of at least three independent experiments.

Therefore, the role of the TFs *CgHAP1* and *CAGL0B03421g* in azole stress tolerance was investigated. The deletion of *CAGL0B03421g* increases *C*. *glabrata* susceptibility to fluconazole, clotrimazole, ketoconazole, and miconazole and more subtly to itraconazole, when compared with the wild-type, whereas no sensitization effect was observed upon *CgHAP1* deletion (Figure 1A). The MIC (Minimum Inhibitory Concentration) values for fluconazole were also determined, revealing a two-fold susceptibility increase upon deletion of *CAGL0B03421g*, while no difference was observed for the *Δcghap1* strain (Table 2). Accordingly, the expression of *CAGL0B03421g* rescues the mutant susceptibility phenotype, and its overexpression in wild-type cells leads to a slight decrease in susceptibility (Figure 1B). The results indicate a role for *CAGL0B03421g* (but not *CgHAP1*—*CAGL0K05841g*) in azole susceptibility phenotype. Since *CAGL0B03421g* is the syntenic *HAP1* ortholog but the designation of *C*. *glabrata* Hap1 is already attributed to *CAGL0K05841g* (the second *HAP1* homolog) and considering that ScHap1 is not known to participate in azole response, the gene *CAGL0B03421g* is henceforth designated *CgMAR1* (Multiple Azole Resistance 1).

### 3.2. Role of CgMar1 in the Transcriptome-Wide Changes Occurring in Response to Fluconazole in C. glabrata

**Figure 2:**
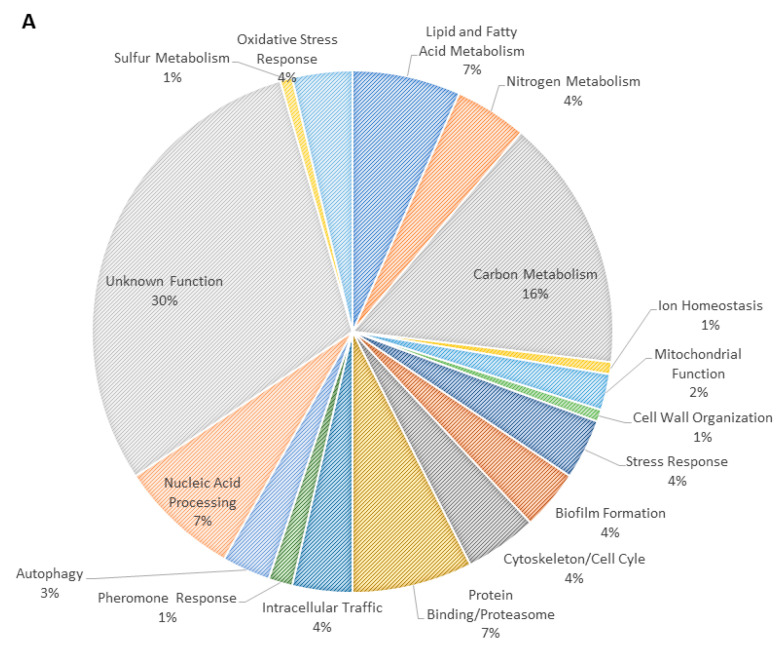

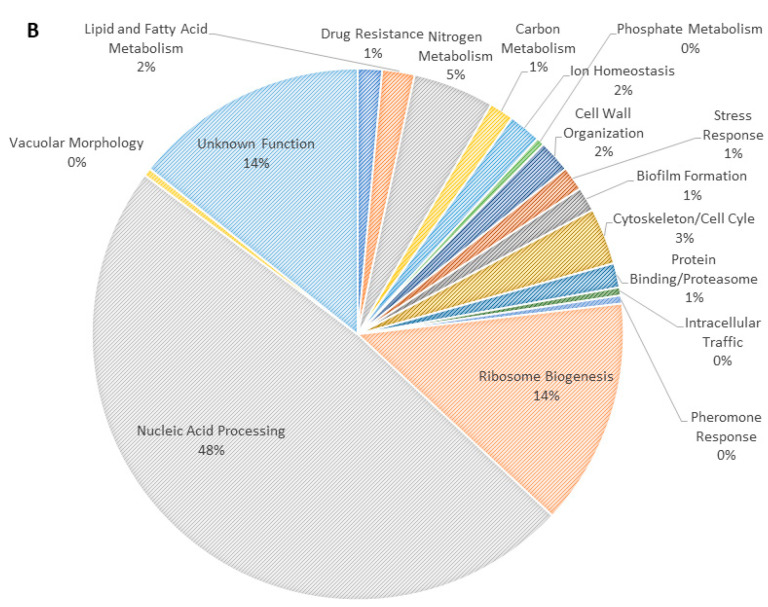
CgMar1-regulated functional groups. Differentially expressed genes in exponential-phase KUE100_*Δcgmar1* cells compared with KUE100 wild-type cells after 1 h of fluconazole exposure. (**A**) Positively CgMar1-regulated genes (downregulated in the mutant strain). (**B**) Negatively CgMar1-regulated genes (upregulated in the mutant strain).

To investigate the role of CgMar1 in fluconazole response, global gene expression changes in *∆cgmar1* cells were assessed through RNA-sequencing and compared with those of wild-type cells. In control conditions, the deletion of *CgMAR1* results in the altered expression of 525 genes: 222 genes show decreased expression, while 303 genes show increased expression in *Δcgmar1* cells when compared with wild-type cells (Table S2). Interestingly, adhesin genes are among those with more prominent expression changes upon *CgMAR1* deletion; however, no changes in biofilm formation were observed for the *Δcgmar1* strain, when compared with the parental wild-type strain, in RPMI medium (Figure S2). Upon fluconazole exposure, the *Δcgmar1* cells presented differential expression of 337 genes: 203 genes showed increased expression and 134 genes showed decreased expression when compared with wild-type cells (Table S3). Among those, 30% were exclusively regulated during antifungal stress and not in control conditions. The most prevalent functional groups of genes putatively activated by CgMar1 (reduced expression in the *Δcgmar1* mutant) during fluconazole stress comprise carbon, nitrogen, and lipid metabolism (Figure 2A), while genes negatively regulated are especially enriched in nucleic acid processing and ribosome biogenesis genes (Figure 2B). Since the antifungal activity of fluconazole leads to defective plasma membrane properties [8,9], the differential regulation of lipid metabolism pathways by CgMar1 is particularly interesting. CgMar1-regulated genes do not include ergosterol biosynthesis but rather sterol transfer genes: *CgLAM1*, *CgLAM5*, and *CgLAM6*. Genes related to sphingolipid (*CgRSB1* and *CgYPC1*) and phospholipid (*CgOPI10* and *CgNTE1*) metabolism as well as the metabolism of carnitine (*CgYAT2*) and fatty acids (*CgHBN1*) are activated by CgMar1.

In the case of stress response genes, the heat-shock protein coding genes *CgHSP78* and *CgSSA3* are activated by CgMar1. The putative heat-shock binding protein encoded by *CgGAC1* is also positively regulated by CgMar1, and its *C*. *albicans* homolog was found to be induced by fluconazole [47]. Moreover, a stress responsive gene (ORF *CAGL0M04763g*) and a gene with a predicted function in cellular response to drug (*CgRTC3*) were found to be activated by CgMar1.

Interestingly, no multidrug resistance transporters were found to be potentially activated by CgMar1. In fact, the MFS transporters *CgTPO1_1* and *CgTPO4*, and the ABC transporter *CgYOR1* were found to have increased expression in the *Δcgmar1* mutant. This hints that *CgMAR1* deletion may disrupt cellular pathways that increase drug susceptibility beyond drug transporter expression.

### 3.3. CgMar1 and CgRsb1 Are Required for Sphingolipid Incorporation in the Plasma Membrane

The previous transcriptomics analysis determined that *CgRSB1* expression may be, directly or indirectly, activated by CgMar1. In both *S*. *cerevisiae* and *C*. *albicans*, Rsb1 homologs (ScRsb1 and CaRta2) are members of the lipid-translocating exporter family, contributing to membrane asymmetry and sphingoid long-chain base release [48,49,50]. In *C*. *glabrata*, transcriptomics studies identified *CgRSB1* as a CgPdr1 target [15,51,52,53]. Although its role in fluconazole resistance remains to be addressed, the *Δcgrsb1* mutant displays a two-fold MIC decrease in fluconazole susceptibility when compared with the wild-type (Table S4).

**Figure 3:**
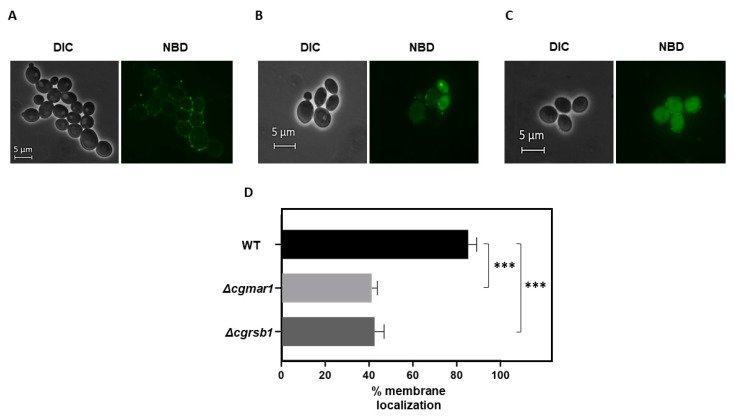
CgMar1 and its activated target CgRsb1 contribute to DHS incorporation in the plasma membrane. (**A**) Representative images of KUE100 *C*. *glabrata* wild-type cell fluorescence upon incorporation of NBD-DHS. (**B**) Representative images of KUE100_*Δcgrsb1 C*. *glabrata* cell fluorescence upon incorporation of NBD-DHS. (**C**) Representative images of KUE100_*Δcgmar1 C*. *glabrata* cell fluorescence upon incorporation of NBD-DHS. (**D**) Percentage of cells showing membrane localization of the NBD-DHS probe. Values are the averages of at least three independent experiments. Error bars represent the corresponding standard deviations. *** *p*-value < 0.001; DIC—Differential Interference Contrast; NBD—Nitrobenzoxadiazole lipid labeling probe.

Therefore, we assessed the subcellular localization of fluorescently labeled dihydrosphingosine (NBD-DHS) in wild-type and *∆cgrsb1* cells. Wild-type cells incorporate DHS in the cell periphery with a dotted pattern, consistent with a lipid raft distribution in the membrane (Figure 3A). Contrarily, the *∆cgrsb1* mutant incorporates less NBD-DHS in the plasma membrane and more inside the cells (Figure 3B). The subcellular localization of NBD-DHS was also assessed in *∆cgmar1* cells, which failed to incorporate NBD-DHS at the plasma membrane (Figure 3C). The quantification of NBD-DHS localization to cell membranes in each population supports these data: wild-type cells were seen to incorporate DHS efficiently (85.3%), while the mutant strains *∆cgmar1* or *∆cgrsb1* were seen to incorporate only 41.3% or 42.6% DHS in the cell membrane, respectively, which represents a two-fold decrease in membrane DHS incorporation (Figure 3D). The evidence suggests that CgRsb1 and its regulator CgMar1 contribute to the incorporation of DHS in the plasma membrane, therefore being involved in plasma membrane organization.

### 3.4. CgMar1 and CgRsb1 Contribute to the Maintenance of Plasma Membrane Permeability and Fluconazole Accumulation of C. glabrata Cells

**Figure 4:**
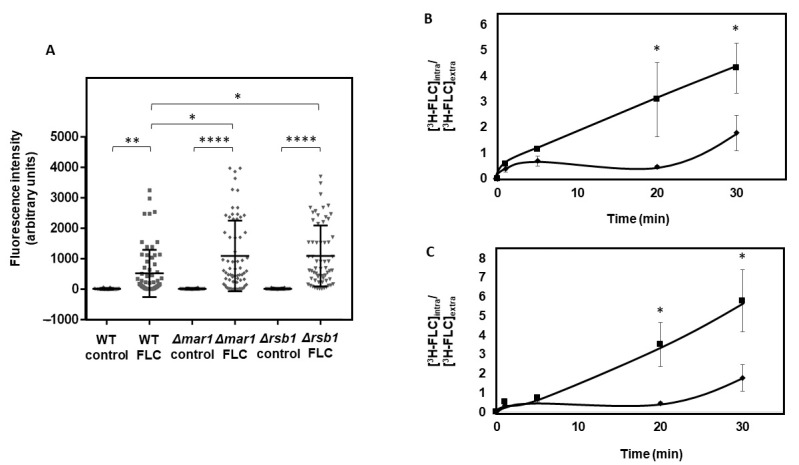
CgMar1 and its activated target CgRsb1 control permeability and drug accumulation in *C*. *glabrata* cells during fluconazole stress. (**A**) Comparison of cell permeability of the KUE100 *C*. *glabrata* wild-type (squares) and derived KUE100_*Δcgmar1* (diamonds) and KUE100_*Δcgrsb1* (triangles) deletion mutant cells under control conditions or after 1 h of fluconazole exposure. The estimation of cell permeability is based on the fluorescence intensity values exhibited by yeast cells upon the passive accumulation of propidium iodide. Error bars represent the corresponding standard deviations. (**B**) Time course accumulation of radiolabeled [^3^H]fluconazole in KUE100 *C*. *glabrata* wild-type (diamonds) and derived KUE100_*Δcgmar1* (squares) deletion mutant cells during cultivation in the presence of unlabeled fluconazole. (**C**) Time course accumulation of radiolabeled [^3^H]fluconazole in KUE100 *C*. *glabrata* wild-type (diamonds) and derived KUE100_*Δcgrsb1* (squares) deletion mutant cells during cultivation in the presence of unlabeled fluconazole. Accumulation values are the averages of at least three independent experiments. Error bars represent the corresponding standard deviations. * *p*-value *<* 0.05, ** *p*-value *<* 0.01, and **** *p*-value *<* 0.0001.

A possible role of CgMar1 and of its target CgRsb1 in the regulation of plasma membrane homeostasis was evaluated via measurements of cell permeability. Upon 1 h of fluconazole exposure, wild-type cell permeability was found to increase significantly (Figure 4A). In comparison, the permeability of *Δcgmar1* cells was not significantly affected in control conditions; however, upon fluconazole stress, it increases to levels higher than the wild-type (Figure 4A). Accordingly, *CgRSB1* deletion results in increased permeability upon fluconazole stress, comparable with the levels registered in the *∆cgmar1* (Figure 4A). This indicates that fluconazole increases *C*. *glabrata* cell permeability and that CgMar1 and CgRsb1 contribute to limiting cell permeabilization during fluconazole exposure.

Consistent with the observed susceptibility and cell permeability phenotypes, the *Δcgmar1* deletion mutant accumulates 4.5-fold more radiolabeled fluconazole than the wild-type only after 30 min of antifungal exposure (Figure 4B). These results suggest that CgMar1 activity mediates *C*. *glabrata* tolerance toward fluconazole by reducing its intracellular accumulation. The accumulation of radiolabeled fluconazole was also assessed in *Δcgrsb1* cells, found to accumulate more intracellular fluconazole than the wild-type and in levels comparable with the *Δcgmar1* mutant (Figure 4C). These results indicate that *CgMAR1* and *CgRSB1* appear to modulate susceptibility to fluconazole by reducing drug intracellular levels.

### 3.5. Identification of Nucleotide Binding Site Recognized by CgMar1 and Association with the CgRSB1 Promoter

To identify CgMar1 DNA recognition sites, the promoters of CgMar1-activated genes during fluconazole stress were searched for enriched motifs using DREME [41]. Excluding TATA-box sequences, five overrepresented consensus sequences were found (Figure S3). From those, we identified two replicates of the CCCCTCC consensus in the *CgRSB1* promoter, in positions −365 to −359 and −357 to −351.

**Figure 5:**
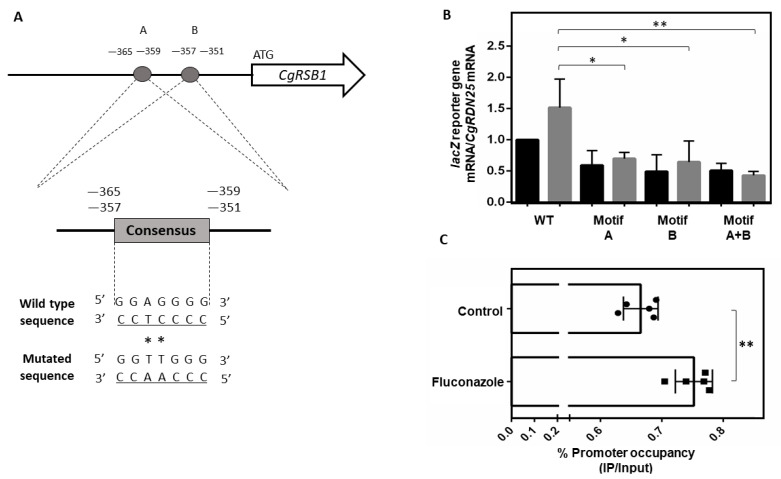
Possible CgMar1 DNA binding sites and association at the *CgRSB1* promoter. (**A**) The putative CgMar1 recognition sequences in the promoter of *CgRSB1* are in the complementary stand. The numbers refer to the position of the consensus site relative to the first ATG of the coding region. The wild-type sequence is shown underlined, with asterisks denoting the base substitutions generated by site-directed mutagenesis. The resulting mutated sequence is shown below. (**B**) Comparison of the variations of lacZ transcript levels determined by RT-PCR in KUE100::URA- *C*. *glabrata* cells harboring the pYEP354_*CgRSB1*prom_lacZ (wild-type promoter) or pYEP354_mutA/B/A+B_*CgRSB1*prom_lacZ (mutated promoters) plasmids under control conditions or after 1 h of fluconazole exposure. Transcript levels of *CgRDN25* were used for normalization. The black bars refer to control conditions, and the grey bars refer to fluconazole exposure. Expression values are the averages from at least three independent experiments. The error bars represent the corresponding standard deviations. (**C**) ChIP-RT-PCR measurements of CgMar1 promoter occupancy at the *CgRSB1* promoter region containing the possible recognition motifs. ChIP experiments were performed using mouse anti-GFP antibody and cultures of KUE100::URA- *C*. *glabrata* cells harboring the pGREG576_PDC1_*CgMAR1* plasmid under control conditions or after 1 h of fluconazole exposure. The amplified amount of DNA was measured by RT-PCR and normalized to the total amount of the sample. Samples from the CgMar1 IP were compared to the input (IP/Input) under each condition. To determine percent occupancy, values were calculated as the ratio of the percent precipitated under fluconazole stress to the percent precipitated under control conditions. Promoter occupancy values are the averages from at least three independent experiments. Error bars represent the corresponding standard deviations. * *p*-value *<* 0.05, ** *p*-value *<* 0.01.

The *CgRSB1* promoter was placed upstream of the lacZ reporter gene and site-directed mutagenesis was used to disrupt each replicate of the CCCCTCC consensus (designated Motif A or Motif B, for simplicity) (Figure 5A). Upon 1 h of fluconazole exposure, lacZ expression increased by 1.5-fold, which is in accordance with the transcriptomics data (Figure 5B). When either Motif A or Motif B was disrupted, lacZ expression from the mutated promoter in control conditions did not significantly change when compared with the wild-type promoter; however, under fluconazole stress, lacZ expression from the mutated promoter was reduced by two-fold compared with the wild-type promoter (Figure 5B). Additionally, when both motifs were concurrently disrupted, a more significant reduction of lacZ expression (three-fold) relative to the wild-type promoter is observed during fluconazole stress (Figure 5B). These results indicate that the two CCCCTCC motifs are required for the activation of *CgRSB1* expression, especially in response to fluconazole.

Subsequently, chromatin immunoprecipitation (ChIP) followed by RT-PCR was used to determine a possible association of CgMar1 to the section of the *CgRSB1* promoter containing these motifs. Indeed, CgMar1 can be detected in association with the promoter of *CgRSB1* in the region containing the two CCCCTCC motifs in control conditions and increases its promoter occupancy upon fluconazole stress (Figure 5C).

## 4. Discussion

In this study, the TF CgMar1 (*CAGL0B03421g*) is characterized as a regulator of fluconazole tolerance in *C*. *glabrata*.

Deletion of *CgMAR1* increases susceptibility to multiple azoles, including fluconazole. The fact that differential susceptibility patterns towards the tested azoles were registered, especially in the case of itraconazole, for which only a subtle effect was observed, is consistent with the notion that azole drugs, although sharing the same primary target, may induce various side-effects and exert different levels of membrane toxicity. Still, the obtained results correlate with a previous screening where the deletion of *CgMAR1* (CAGL0B03421g) was found to increase fluconazole susceptibility [54]. On the other hand, the deletion of the *CgMAR1* homolog, *CgHAP1*, leads to no phenotypic changes in relation to most azoles while inducing a modest increase in the resistance to fluconazole and clotrimazole. It would be interesting to investigate why CgHap1 has this effect, which may eventually be linked to its role as a heme response regulator, with heme being a crucial prosthetic group of the azole target protein, Erg11.

RNA-seq based transcriptomics revealed that CgMar1 regulates distinct cellular processes; however, it does not activate MDR transporters. Despite being a *ScHAP1* homolog, CgMar1 presents a very limited activated regulon overlap with it (Figure S4). This appears to be consistent with the dissimilar CgMar1 phenotype and its putative recognition motifs predicted in our study being distinct from the established ScHap1 binding site (two CGG triplets). Among others, CgMar1 regulates genes involved in plasma membrane lipid biosynthesis and incorporation pathways, which have been correlated with azole resistance in *C*. *albicans*. In that species, an azole-resistant isolate showed altered sterol and phosphatidylcholine:phosphatidylethanolamine ratio [55]. Furthermore, response to fluconazole comprises the upregulation of sphingolipid biosynthesis genes and an altered sphingolipid composition [56]. *C*. *albicans* azole susceptibility was also associated with defective phospholipid metabolism, increased membrane fluidity, and accumulation of fluconazole [57]. Our results support similar responses in *C*. *glabrata*, as fluconazole exposure increased plasma membrane permeability. Both CgMar1 and its activated target CgRsb1 are required to control permeability levels and intracellular fluconazole accumulation. Interestingly, CgMar1 negatively affects the expression of another gene from the same lipid exporter family, *CgRTA1*, under control conditions. This raises the question of whether each gene may have opposite functions, for example, the encoded proteins acting as flippase/floppase. The differential activity of the multiple genes from this family may provide clarifications on membrane homeostasis.

CgMar1 and CgRsb1 contribute to the correct incorporation of sphingolipid molecules in the membrane. In *C*. *albicans*, the *CaRTA2* homolog contributes to membrane homeostasis [50] and is upregulated in clinical azole-resistant isolates and in an induced fluconazole-resistant strain [58,59]. It localizes to lipid rafts and is required for the association of proteins to such rafts, including CaErg11 [60]. Furthermore, the lipid translocase *CaRTA3* is required to maintain phosphatidylcholine asymmetry in the plasma membrane [61], is upregulated upon ketoconazole exposure [62], and is coordinately upregulated with *CaCDR1* and *CaCDR2* during fluconazole stress [63]. This pattern is also observed in *C*. *glabrata*: CgPdr1 upregulates not only *CgCDR1* but also *CgRSB1*, associating to their promoters [15,51,52,53]. The relevance of lipid flippases in azole resistance has also been demonstrated in *C*. *neoformans* [64]. This study lifts the veil on a possible involvement of CgMar1 as an additional regulator of CgRsb1, therefore justifying the correct sphingolipid incorporation in the plasma membrane and its correlation with *C*. *glabrata* susceptibility to fluconazole. However, given that membrane homeostasis impacts multiple cellular processes, including the correct sorting and function of plasma membrane proteins, a definitive mode of action for CgMar1 or CgRsb1 cannot be established yet. It is possible that these genes impact azole susceptibility indirectly by contributing to the maintenance of membrane homeostasis and, consequently, the correct action of azole resistance determinants, such as MDR transporters.

Sphingolipids also participate in antifungal resistance through interactions with ergosterol. Matched susceptible and resistant *C*. *albicans* clinical isolates revealed gradual changes in mannosylinositolphosphorylceramides and ergosterol [65]. Moreover, both ergosterol and the toxic sterol DMCDD integrate into the cell membrane through interaction with sphingolipids [66]. Additionally, the lipid composition of the plasma membrane can influence the activity of MDR transporters. Imbalances between membrane sphingolipids and sterols impair the sorting and functioning of ABC transporters to lipid rafts [67]. Moreover, fluconazole decreases ordered lipid domains containing integral proteins and protein content [68]. Future studies could investigate imbalanced membrane compositions and the relative contributions for increased import vs. decreased transport of drugs. The increased intracellular fluconazole accumulation observed when *CgMAR1* or *CgRSB1* are deleted in *C*. *glabrata* should not be unequivocally correlated solely with antifungal import at this time as it may reflect (partially or totally) a contribution from reduced azole export prompted by defective membrane composition or structure.

## Figures and Tables

**Table 1:** *C*. *glabrata* strains used in this study.

Strain	Genotype	Parent	Reference
KUE100	Wild type	CBS138	[25]
KUE100_*Δcgmar1*	*Δcgmar1*	KUE100	This study
KUE100_*Δcghap1*	*Δcghap*	KUE100	This study
KUE100_*Δcgrsb1*	*Δcgrsb1*	KUE100	This study
KUE100::URA-	*Δcgura3*	KUE100	[26,27]
KUE100_*Δcgmar1*::URA-	*Δcgmar1Δcgura3*	KUE100_*Δcgmar1*	This study
KUE100::URA- + pGREG576_PDC1_*CgMAR1*	*Δcgura3* + *CgMAR1*	KUE100::URA-	This study
KUE100_*Δcgmar1*::URA- + pGREG576_PDC1_*CgMAR1*	*Δcgmar1Δcgura3* + *CgMAR1*	KUE100_*Δcgmar1*::URA-	This study
KUE100::URA- + pYEP354_*CgRSB1*prom_lacZ	*Δcgura3* + lacZ	KUE100::URA-	This study
KUE100::URA- + pYEP354_mutA/B/A+B_*CgRSB1*prom_lacZ	*Δcgura3* + lacZ	KUE100::URA-	This study

**Table 2:** Fluconazole susceptibility profiles determined by MIC values in deletion mutants for *CgMAR1* and *CgHAP1* and the corresponding parental strain (WT).

Strain	Fluconazole MIC (mg/L)
WT	16
*Δcgmar1*	8
*Δcghap1*	16

## Data Availability

RNA-sequencing data sets were deposited at the Gene Expression Omnibus, NCBI database, with the reference number GSE163158. All other data are available from the corresponding author upon reasonable request.

## Supplementary Materials

The following supporting information can be downloaded at https://www.mdpi.com/article/10.3390/jof8010061/s1, Figure S1: Dot plot depicting the alignment results for the *C. glabrata* protein encoded by ORF *CAGL0B03421g*. (A) Blastp alignment result between the proteins encoded by *CAGL0B03421g* (*C. glabrata*) and *CaZCF20* (*C. albicans*). Conserved domains and their positions are indicated at the bottom of the dot plot. (B) Blastp alignment result between the proteins encoded by *CAGL0B03421g* (*C. glabrata*) and *CaMRR1* (*C. albicans*). Conserved domains and their positions are indicated at the bottom of the dot plot; Figure S2: Assessment of 24 h biofilm formation was performed by Violet Crystal in microtiter plates of the deletion mutant for *CgMAR1* and the corresponding parental strain.; Figure S3: Consensus found to be overrepresented in the promoters of CgMar1-activated genes, as found by the DREME tool. The sequences shown correspond to the motifs outputted by DREME, excluding TATA box motifs; Figure S4: Comparison of the activated regulons between ScHap1 (blue) and CgMar1 (yellow), irrespective of experimental condition. The regulon of ScHap1 was retrieved from the PathoYeastract database and the *C. glabrata* homologs used for the comparison; Table S1: List of primers used in this study; Table S2: Differentially expressed genes in *∆cgmar1* control/WT control; Table S3: Differentially expressed genes in *∆cgmar1* FLC/WT FLC; Table S4: Fluconazole susceptibility profiles determined by MIC values in the deletion mutant for *CgRSB1* and the corresponding parental strain.
