# Supplementary material for: Characterization of the Candida glabrata Transcription Factor CgMar1: Role in Azole Susceptibility
Source: J Fungi (Basel). 2022 Jan 7;8(1):61. doi: 10.3390/jof8010061 (PMC8779156; doi:10.3390/jof8010061)
Supplement: Supplementary file 1 [file jof-08-00061-s001.zip › Figures S1-S4.pdf]

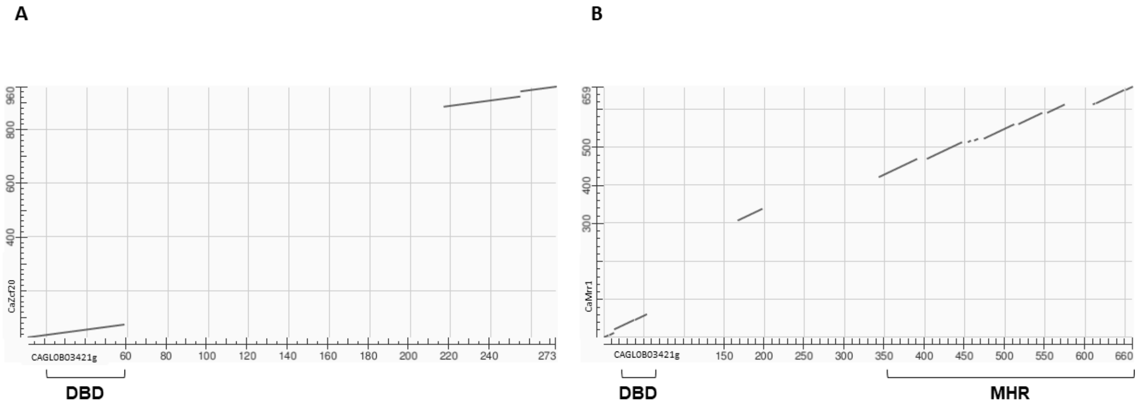

Figure S1: Dot plot depicting the alignment results for the *C. glabrata* protein encoded by ORF *CAGL0B03421g*. (A) Blastp alignment result between the proteins encoded by *CAGL0B03421g* (*C. glabrata*) and *CaZCF20* (*C. albicans*). Conserved domains and their positions are indicated at the bottom of the dot plot. (B) Blastp alignment result between the proteins encoded by *CAGL0B03421g* (*C. glabrata*) and *CaMRR1* (*C. albicans*). Conserved domains and their positions are indicated at the bottom of the dot plot.

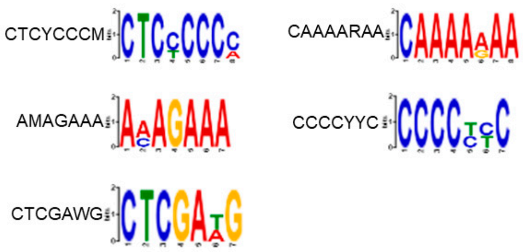

Figure S2: Assessment of 24 h biofilm formation was performed by Violet Crystal in microtiter plates of the deletion mutant for *CgMAR1* and the corresponding parental strain.

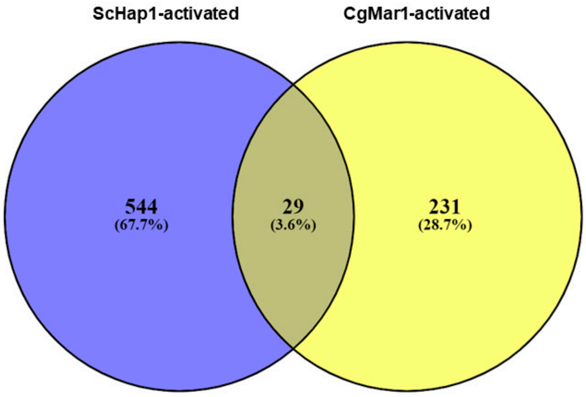

Figure S3: Consensus found to be overrepresented in the promoters of CgMar1-activated genes, as found by the DREME tool. The sequences shown correspond to the motifs outputted by DREME, excluding TATA box motifs.

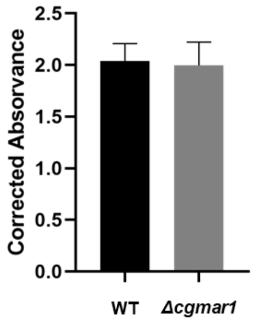

Figure S4: Comparison of the activated regulons between ScHap1 (blue) and CgMar1 (yellow), irrespective of experimental condition. The regulon of ScHap1 was retrieved from the PathoYeastract database and the *C. glabrata* homologs used for the comparison
