# Supplementary material for: Characterization of the Candida glabrata Transcription Factor CgMar1: Role in Azole Susceptibility
Source: J Fungi (Basel). 2022 Jan 7;8(1):61. doi: 10.3390/jof8010061 (PMC8779156; doi:10.3390/jof8010061)
Supplement: Supplementary file 1 [file jof-08-00061-s001.zip › Table_S1.pdf]

Supplementary Table S1 – List of primers used in this study.

Primer	Sequence
Deletion of *CgMAR1*	5’-TTTACATCTCTACTAGCTGTTTCGATTTAAGTATTCCGCTATACTCACTGTACCCGGCCGCTGATCACG-3’<br>5’-AATTGTATTATATATATATATGACTGTGGAAAAATTAAATACACAAACATAACAAATGCCATCGTGAGGCTGG-3’
Confirmation of *CgMAR1* deletion	5’-CCACTACAACAATACCAGGA-3’<br>5’-GAGTTCGTCATAATCGTGTTC-3’
Deletion of *CgHAP1*	5’-AAAGAGCCATTTTATATTGCAAAGAGAGGAACAATAATCTACGGATCTAAATCCTGGGCCGCTGATCACG-3’<br>5’-CACAATTAAATATTATATATCCTTTCTAAAGCCTGATTATATTCCACTAAGAATTACATCGTGAGGCTGG-3’
Confirmation of *CgHAP1* deletion	5’- GCGATGTATGCGTTGCCT-3’<br>5’-GTCACATCGGTCAGAGAGT-3’
Deletion of *CgRSB1*	5’-TGATGGATGAGGTGGTGGAAAAATTCACGTTTGATCCACTCTCGTATTATTCGGGGGGCCGCTGATCACG-3’<br>5’-AAATGAGATTAAAGCCGAGTTCCTTGATTTTGATGAAAAGTTTTTGCCATCCTTTACATCGTGAGGCTGG-3’
Confirmation of *CgRSB1* deletion	5’-CCCAAGCTGAGGTTCAATG-3’<br>5’-CATGGAGGCAAGCTGAAT-3’
Cloning of *CgMAR1* in pGREG576	5’–GAATTCGATATCAAGCTTATCGATACCGTCGACA*ATGAGTACCACTACAACAATACC*-3’<br>5’–GCGTGACATAACTAATTACATGACTCGAGGTCGAC*CTACTCCCAGATTGAGCCAATG*-3’
Cloning of *CgPDC1* promoter in pGREG576_*CgMAR1*	5’-TTAACCCTCACTAAAGGGAACAAAAGCTGGAGCTC*AGCATTTTTATACACGTTTTAC*-3’<br>5’-GAAAAGTTCTTCTCCTTTACTCATACTAGTGCGGC*TGTTAATGTTTTTTGGCAATTG*-3’
Cloning of *CgURA3* gRNA in pV1382	5’-GATCGACCGGCCAAGGTATCGTCACG-3’<br>5’-AAAACGTGACGATACCTTGGCCGGTC-3’
Cloning of *CgRSB1* promoter in pYEP354	5’-CCGGAATTCCGTACACAAGCAGCTAGGTAAT-3’<br>5’-AACTGCAGCTCATCCATCATTAGTTATT-3’
Mutagenesis of possible	5'-CTCAGAAATTGGGGTTGGGGGGGAGGGATG-3'<br>5'-CATCCCTCCCCCCCAACCCCAATTTCTGAG-3'

CgMar1 consensus A	
Mutagenesis of possible CgMar1 consensus B	5'-GAAATTGGGGGAGGGGGGTTGGGATGAGGTGGAAGTG-3'<br>5'-CACTTCCACCTCATCCCAACCCCCCTCCCCCAATTTC-3'
Mutagenesis of possible CgMar1 consensus A + consensus B	5’-CTCAGAAATTGGGGTTGGGGGGTTGGGATG-3’<br>5’-CATCCCAACCCCCCAACCCCAATTTCTGAG-3’
LacZ expression measurement	5’-TGGCTGGAGTGCGATCTTC-3’<br>5’-CGTGCATCTGCCAGTTTGAC-3’
CgRDN25 expression measurement	5’-AACAACTCACCGGCCGAAT-3’<br>5’-CAAGCGTGTTACCTATACTCCGCCGTCA-3’
Quantification of consensus A+B by ChIP-RT-PCR	5’- AGGGCATTGATGAGATCTGG-3’<br>5’- GTCTGGCTTGCTTCCTTGAC-3’
