# Supplementary material for: Characterization of the Candida glabrata Transcription Factor CgMar1: Role in Azole Susceptibility
Source: J Fungi (Basel). 2022 Jan 7;8(1):61. doi: 10.3390/jof8010061 (PMC8779156; doi:10.3390/jof8010061)
Supplement: Supplementary file 1 [file jof-08-00061-s001.zip › Table_S4.pdf]

Supplementary Table S4 – Fluconazole susceptibility profiles determined by MIC values in the deletion mutant for *CgRSB1* and the corresponding parental strain.

**Strain**	**Fluconazole MIC (mg/L)**
WT	16
Δcgrsb1	8
